# Leptin induces osteocalcin expression in ATDC5 cells through activation of the MAPK-ERK1/2 signaling pathway

**DOI:** 10.18632/oncotarget.11578

**Published:** 2016-08-24

**Authors:** Yingchao Han, Guanghui Xu, Jingjie Zhang, Meijun Yan, Xinhua Li, Bin Ma, Lili Jun, Shan-Jin Wang, Jun Tan

**Affiliations:** ^1^ Department of Spine Surgery, Shanghai East Hospital, Tongji University, School of Medicine, Shanghai, 200120, China; ^2^ Department of Orthopedics, Shanghai Zhabei District Central hospital, Zhonghuaxin Road Zhabei District, Shanghai, 200070, China

**Keywords:** leptin, osteocalcin, ATDC5, growth plate, chondrocyte

## Abstract

Both leptin and osteocalcin have been found to affect growth-plate cartilage development through regulation of the physiologic processes of endochondral bone formation. Leptin mediates bone development and osteocalcin secreted in the late stage of osteoblast differentiation. The relationship between leptin and osteocalcin expression in the chondrogenic cells line is still not clear. Thus, the aim of this study was to explore the effect of leptin on the expression of osteocalcin in chondrocytes. We used clonal mouse chondrogenic ATDC5 cells to investigate the relationship between leptin and osteocalcin. We found that both leptin and osteocalcin expression were dynamically expressed during ATDC5 cell differentiation from 4 to 21 days. We also found that leptin significantly upregulated osteocalcin mRNA and protein levels 24 h after leptin stimulation. However, different concentrations and exposure times of osteocalcin did not affect the levels of leptin protein. Furthermore, we confirmed that leptin augmented the phosphorylation of extracellular signal-regulated kinase 1/2 (ERK1/2) in a time-dependent manner but not p38 or AKT. Inhibition of pERK1/2 expression by a specific ERK1/2 inhibitor U0126 and a special small interfering RNA attenuated levels of leptin-induced osteocalcin expression, indicating that ERK1/2 mediates, in part, the effects of leptin on osteocalcin. Taken together, our results suggest that leptin regulates the expression of osteocalcin in growth plate chondrocytes via the ERK1/2 signaling pathway, while there is no effect on the phosphorylation of either p38 or AKT.

## INTRODUCTION

The growth and maintenance of the mammalian skeleton is a product of developmental and postnatal processes that occur throughout life. During endochondral ossification, cartilage serves as a template for bone development. The growth plate and its primary cell type, the chondrocyte, are integral to endochondral ossification and thus the linear growth of the long bones [1]. During differentiation, chondrocytes secrete extracellular matrix molecules characteristic of cartilage, such as collagen type II and X and aggrecan. This process is controlled by many types of endocrine factors, growth factors, thyroid hormone, and leptin [2, 3].

Bone tissue is composed of cells and mineralized extracellular matrix. The latter consists of hydroxyapatite crystals that load a stress-bearing organic matrix of collagen fibers and non-collagenous proteins, such as osteocalcin, osteopontin, osteonectin, and bone sialoproteins. Osteocalcin, the most abundant non-collagenous bone matrix protein [4], is a small g-carboxyglutamate protein preferentially expressed by osteoblasts that is able to bind calcium ions. The exact role of osteocalcin in bone is not yet completely understood, but it is clearly involved in regulating bone mineralization and bone turnover [5]. Currently, serum osteocalcin levels are used to evaluate bone metabolism, as a bone formation marker, and it is thought to be a more sensitive marker for bone metabolism than serum alkaline phosphatase activity [6, 7].

Leptin, produced primarily by adipocytes, is a pleiotropic hormone [8]. It became apparent recently that leptin plays a role in bone remolding by mobilizing leptin receptors expressed by cells in bones [9, 10]. Leptin also plays an important role in the regulation of endochondral bone formation, proliferation, and mineralization. It is induced in chondrocyte apoptosis and osteoarthritis [11–14]. Some studies reported that leptin tends to reduce bone fragility, can contribute to high bone mass [15, 16], and it can increase osteocalcin release from osteoblasts [17].

The mitogen-activated protein kinase (MAPK) group of signal transduction pathways transmits extracellular signals that stimulate mitogenic and stress responses. Each MAPK cascade is composed of several protein kinases that specifically phosphorylate and activate each other in a hierarchical manner, thereby forming a complex signaling network. Four distinct MAPK cascades have been recognized: extracellular signal-regulated kinase 1/2 (ERK1/2), c-Jun N-terminal kinase, p38MAPK, and big mitogen-activated kinase 1, known also as ERK5 [18]. Our previous study showed that leptin regulates the expression of ERs (estrogen receptor) in growth plate chondrocytes via the ERK signaling pathway [19]. Leptin can activate many signaling pathways involving the Janus kinase/signal transducer and activator of transcription (JAK/STAT), as well as phosphatidylinositol 3-kinase and MAPK, to regulate chondrocyte differentiation [20–24]. However, their involvement in leptin induction of osteocalcin has not been investigated.

The main aim of this study was to investigate the effect of leptin on the expression of osteocalcin by using micromass culture of the mouse chondrogenic ATDC5 cell line. This micromass culture is a reliable *in vitro* model for investigating temporal modulation of factors that coordinate chondrocyte differentiation and chondrogenesis [25].

## RESULTS

### Differentiation of ATDC5 cells

**Figure 1:**
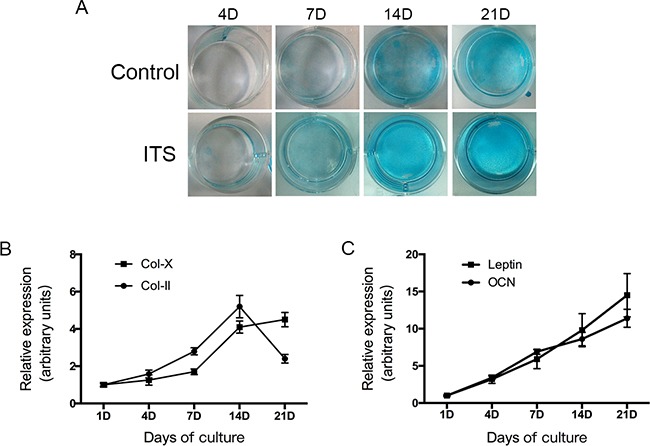
ATDC5 cell differentiation in culture ATDC5 cells were cultured in six-well plates at a density of 6×104/well in DMEM/F12 medium containing 5% FBS and 1% ITS for 1,4,7,14 and 21 days. The cells then washed with PBS twice, fixed with 4% Paraformaldehyde then stained with 1% Alcian blue for 30 mins. Relative expression of collagen II, X, leptin and osteocalcin mRNA was determined by real-time PCR.

We evaluated whether treatment with ITS stimulates formation of cartilage nodules in ATDC5 cells. Cells were treated with ITS for 21 days. Figure 1A shows that matrix proteoglycan synthesis was verified by nodules stained with Alcian blue dye; staining intensities in ATDC5 cells cultured with ITS gradually increased in a time-dependent manner from 4 to 21 days. The expression of extracellular matrix genes, including those for type II and type X collagen, was used to characterize the chondrogenic differentiation of ATDC5 cells.

We then evaluated the expression of chondrogenic differentiation markers by using real-time PCR. The differentiation of mesenchymal cells into chondrocytes was indicated by an increase in the mRNA transcript coding for collagen type II expression in ATDC5 cells after a single day in the differentiation medium. As Figure 1B shows, collagen II mRNA expression markedly increased between days 7 and 14, which indicates early-stage differentiation of chondrocytes. The level of collagen type X mRNA gradually increased from day 7 onwards and maintained high levels between days 14 and 21, indicating late-stage differentiation of chondrocytes. On day 21, ATDC5 cells primarily expressed type X collagen instead of type II collagen.

These results show that undifferentiated ATDC5 cells differentiate in culture into proliferative chondrocytes and then to hypertrophic chondrocytes, validating the use of this cell system as an *in vitro* model to study chondrocyte differentiation.

### Expression of leptin and osteocalcin in ATDC5 cells

We also examined the changes of leptin and osteocalcin during ATDC5 cell differentiation in culture. The results of real time PCR confirmed that both leptin and osteocalcin mRNA were dynamically expressed in ATDC5 cells during all of the differentiation phases (Figure 1C). Both leptin and osteocalcin mRNA levels were significantly increased during the progression of chondrogenic differentiation (days 4–21).

### Effect of leptin on osteocalcin expression in ATDC5 cells

**Figure 2:**
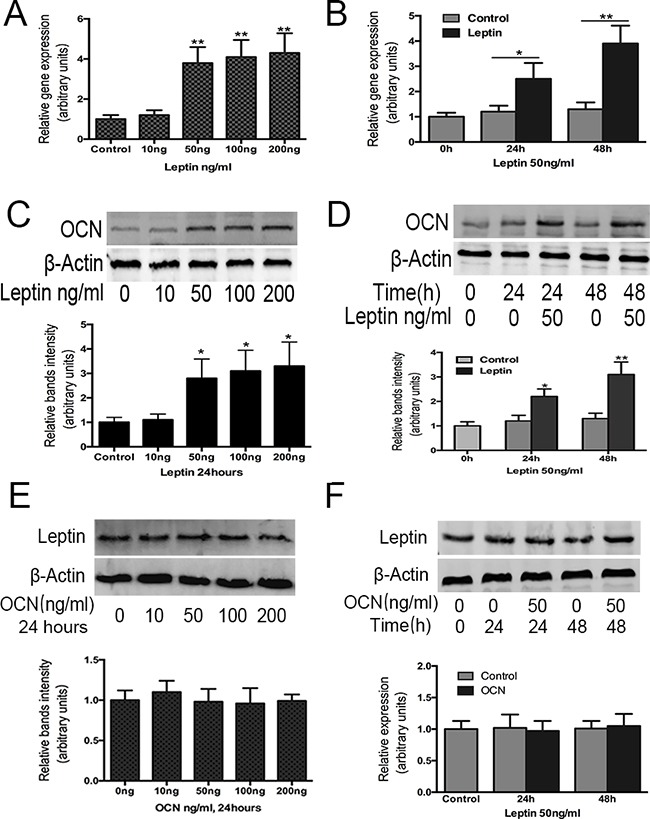
Effect of leptin on osteocalcin mRNA, protein expression and effect of osteocalcin on leptin protein expression ATDC5 cell were cultured in DMEM/F12 containing 5% FBS and 1%ITS in six-well plates at a density of 6×10^4^/well for 14 days. Cultured cells were then treated with and without leptin or OCN at 10,50,100,200ng/ml for 48h. OCN **A, C.** gene and protein expression were analyzed by real-time PCR and western-blot, were normalized against β-actin and compared to control group. Leptin protein expression **E.** was also analyzed by western-blot. Cultured cells were also treated with and without leptin or OCN 50ng/ml for 24h and 48h. OCN gene, protein expression **B, D.** and leptin protein expression **F.** were analyzed by RT-PCR and western-blot. Data represent mean±SEM from triplicate samples in three independent experiments. *P<0.05 versus control (0h), **P<0.01 versus control(0h).

To determine whether leptin can induce the up regulation of osteocalcin, ATDC5 cells were cultured with increasing concentrations (0, 10, 50, 100, 200 ng/mL) of exogenous leptin for 48h from day 14 to day 16. Increasing concentrations of leptin resulted in progressive up regulation of mRNA coding for osteocalcin, with the concentration of 200 ng/mL being the most effective (Figure 2A). Thus, osteocalcin mRNA expression increased with leptin treatment in a dose-dependent manner.

The timing of leptin treatment on osteocalcin gene expression in ATDC5 cells was then studied using leptin treatments of 50 ng/mL for 24 h and 48 h, respectively. After 24 and 48 h, osteocalcin mRNA expression was significantly increased by leptin compared with the control group and with baseline levels (Figure 2B).

Next, we examined whether the changes in osteocalcin mRNA expression were followed by a corresponding increase in protein level in cells treated with leptin for 48 h at varying doses (0, 10, 50, 100, 200 ng) (Figure 2C) and when treated with leptin (50 ng) for varying durations (24 and 48 h) (Figure 2D). Similar to the mRNA expression pattern, osteocalcin protein levels were significantly increased in the presence of leptin after 24 and 48 h of treatment compared with the baseline control. These results confirmed that osteocalcin was induced by leptin treatment and osteocalcin protein levels increased in a time-dependent manner, demonstrating that the regulation of osteocalcin gene expression by leptin correlates with changes at the protein level.

### Effect of osteocalcin on leptin expression in ATDC5 cells

We also examined whether osteocalcin regulated leptin expression. ATDC5 cells were treated with osteocalcin from 0 to 200 ng for 48 h (Figure 2E) and were also treated with osteocalcin (50 ng) for 24 h and 48 h (Figure 2F). However, the level of leptin protein expression was not changed after the osteocalcin treatment compared with the level of protein expression before the treatment.

### Role of ERK, p38, and AKT signaling in leptin-induced osteocalcin expression

**Figure 3:**
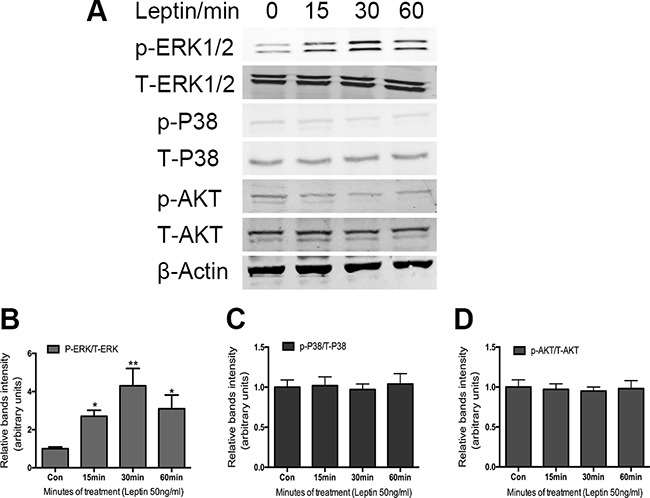
Effect of leptin on the activation of MAPK-AKT phosphorylation in ATDC5 cells Cells were cultured in DMEM/F12 containing 5%FBS and ITS in 6-well plates at a density of 6×10^4^/well for 14 days. Cultured cells were treated with and without 50ng/ml leptin for indicated times. Cell extracts were analyzed by western blot analysis using specific antibodies against the phosphor rylated forms of the protein. Bands **A.** show representative Western-blots, whereas graphs **B, C, D.** show normalized data. Data represent mean±SEM from triplicate samples in three independent experiments. *P<0.05 versus control, **P<0.01 versus control.

MAP kinases have been shown to contribute to a diverse portfolio of cellular events, including cellular differentiation [18], proliferation [26], migration [27], and apoptosis [28]. We sought to determine whether MAP kinase cascades play a role in the chondrogenic response of ATDC5 cells. Previous research showed that the ERK pathway plays an important role in chondrocyte differentiation. To verify whether ERK can be activated by leptin treatment, we measured the expression of p-ERK in the ATDC5 cells following leptin treatment (50 ng/mL) at a series of time points. After 15 min, p-ERK was significantly increased by 3-fold in response to leptin stimulation compared with the control (Figure 3A).

To determine whether p38 and AKT also play a role in the leptin-induced osteocalcin expression, we further examined the effect of leptin (50 ng/mL) on p38 and AKT phosphorylation. As shown in Figure 3A, there is no effect on the phosphorylation of either p38 or AKT following leptin treatment in the ATDC5 cells, suggesting that p38 and AKT do not play a role in leptin-induced osteocalcin expression.

### Role of ERK signaling in leptin-induced osteocalcin expression

**Figure 4:**
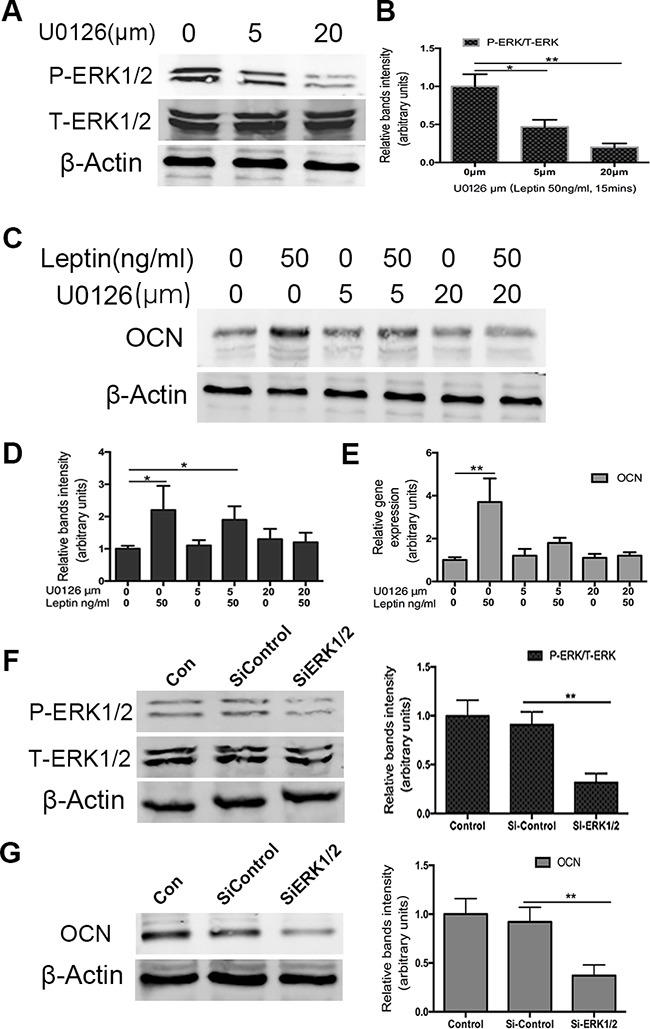
Effect of U0126 and siRNA ERK1/2 on leptin-induced OCN expression Cells were cultured in DMEM/F12 containing 5%FBS and ITS in 6-well plates at a density of 6×104/well for 14 days. Cultured cells were treated with U0126 (5 and 20μm) for 60min and then treated with 50ng/ml leptin 15mins. P-ERK1/2 and ERK1/2 protein levels were analyzed by western blot. Bands **A.** show representative Western-blots, whereas graphs **B.** show normalized data. OCN gene and protein levels were analyzed by RT-PCR **E.** and western blot **C,D.** using specific antibodies as indicated. The silence ERK1/2 effect was confirmed by western blot **F.** After silence ERK1/2, OCN protein expression was also tested by western blot **G.** Data represent mean±SEM from triplicate samples in three independent experiments. *P<0.05 versus control, **P<0.01 versus control.

To assess whether osteocalcin expression was stimulated through the ERK pathway, U0126 was used to inhibit the phosphorylation of ERK1/2. We first used U0126 to assess the inhibitory effects of phosphorylation of ERK1/2 by western blotting. As shown in Figure 4A, 5 μM and 20 μM U0126 significantly reduced the expression of p-ERK1/2. Then, we investigated the inhibitory effects of U0126 on leptin-induced osteocalcin. ATDC5 cells were pretreated with U0126 (5 or 20 μM) for 1 h and then incubated with 50ng/mL leptin for 48 h. Using RT-PCR and western blotting, we found that U0126 significantly blocked the effect of leptin on the up regulation of osteocalcin protein (Figure 4C) and mRNA (Figure 4E). Complete inhibition was observed at the lowest concentration of U0126 used (5 μM). To further investigate whether leptin-induced expression of the osteocalcin gene was mediated through ERK, ATDC5 cells were pretreated with ERK1/2 SiRNA and then incubated with 50ng/mL leptin for 48 h. In response to ERK1/2 SiRNA, osteocalcin protein expression was very low compared with the control group. Taken together, these results strongly suggest that the up regulation of osteocalcin by leptin is mediated by ERK1/2 phosphorylation.

## DISCUSSION

Chondrogenesis is an important biological event involved in tissue patterning, skeletal development, and endochondral ossification [1]. Chondrogenesis begins with the condensation of multipotent mesenchymal cells; these cells proliferate and differentiate into chondrocytes. Many factors influence the longitudinal growth of bone [29]. Nutritional, hormonal, and mechanical factors all affect cell proliferation, hypertrophy, and death, as well as bone formation.

The concentration of osteocalcin in the circulation reflects the amount of bone formation. The function of osteocalcin in bone is thought to be dictated by its structure. Immunolocalization studies show that the protein of osteocalcin is distributed throughout the mineralized regions of bone matrix, dentine, and calcified cartilage in rats [30]. *In vitro* growth of hydroxyapatite is inhibited by low concentrations of osteocalcin, which suggests that this protein's function is related to the control of crystal morphology [31, 32]. In the bone of all species studied, osteocalcin first appears coincident with the onset of mineralization *in utero*, and its levels increase in tandem with hydroxyapatite deposition during skeletal growth.

Observational and interventional studies in animals and humans have shown that leptin plays multiple important roles in bone growth and bone metabolism through both central and peripheral pathways [33, 34]. Leptin deficiency in humans and mice leads to phenotypic abnormalities in skeletal development, the endocrine system, the immune system, and the sympathetic nervous system [35–37]. Signal transduction by leptin has been studied extensively in numerous experimental systems, *in vitro* and *in vivo*[38, 39]. The authors previously found that JAK2, phosphatidylinositol 3-kinase, p38, and ERK were activated in the ATDC5 cell model during synergistic stimulation of collagen type II by leptin and interleukin-1 [40]. However, whether osteocalcin expression can be regulated by leptin through MAPK has not been studied.

In our study, we analyzed the effect of leptin on chondrogenic differentiation *in vitro* using a murine ATDC5 chondrocytic cell line. We found that leptin increased the expression of osteocalcin in a time- and dose-dependent manner. At the same time, leptin increased the phosphorylation of ERK1/2, and the specific inhibition of ERK1/2 abolished the effect of leptin on osteocalcin expression.

To specifically examine the effect of leptin on differentiation at the molecular level, we first studied mRNA expression of type II and X collagen, markers of proliferative and hypertrophic chondrocytes, respectively. We found in ATDC5 cells that type II collagen mRNA expression initially increased and then decreased during differentiation, and type II collagen mRNA expression was apparently replaced by type X collagen during the final time points.

We confirmed that the expression of leptin and osteocalcin can be up regulated during chondrocytic differentiation in ATDC5 cells. In the present study, leptin was added to the culture medium from day 14, a time when ATDC5 cells express a large amount of type II and type X collagen mRNA, during the middle stage between proliferative and prehypertrophic chondrocytes. The dose of leptin we selected was based on a previous study in which 50ng/mL leptin was used to demonstrate pathways in the differentiation of ATDC5 cells [20].

These data also show a central role of the up regulation of ERK in up regulating osteocalcin expression in response to leptin treatment. The ERK1/2 kinases have been shown to be crucially involved in chondrocyte differentiation because inhibition of ERK signaling with U0126 blocks insulin-induced chondrogenesis [41]. In the present study, we found that U0126 inhibited osteocalcin protein expression of osteocalcin 48 h after leptin stimulation, indicating that activation of ERK1/2 is essential for leptin-induced osteocalcin expression in chondrogenic differentiation of ATDC5 cells.

In conclusion, we demonstrated that both osteocalcin and leptin expression in chondrocytes increases over the duration of the cell differentiation process, and osteocalcin expression can be up regulated by leptin in a time- and dose-dependent manner. Furthermore, our findings suggest that osteocalcin up regulation by leptin is mediated through ERK signaling. The results of our research may have significant implications in understanding the mechanisms of longitudinal bone growth, but further studies are needed to confirm our findings and to define the other possible mechanisms involved.

## MATERIALS AND METHODS

### Reagents

Dulbecco's modified Eagle's medium (DMEM)/Ham's F-12 medium (DMEM/F12; 1:1 mixture), and penicillin-streptomycin solution were obtained from Invetrigen. Insulin, transferrin, sodium selenite (ITS), and Alcian blue 8GX were purchased from Sigma. The primary antibodies against β-actin, phospho-ERK1/2 (Thr202/Thr204), total-ERK1/2, phospho-p38 MAPK (Thr180/Tyr182), anti-p38 MAPK, phospho-AKT (Ser473), AKT antibodies, and MEK1/2 inhibitor U0126 were purchased from Cell Signaling Technology. The primary antibodies against leptin and osteocalcin antibodies were purchased from Abcam company. Fetal bovine serum (FBS) was obtained from Gibco. Recombinant mouse leptin was obtained from R&D. Recombinant mouse osteocalcin was obtained from Cusabio.

### Cell culture and treatments

ATDC5 cells were cultured in DMEM/F12 medium supplemented with 5% FBS, 100 U/mL penicillin, and 100 μg/mL streptomycin in a humidified incubator with 95% air and 5% CO_2_ at 37°C. ATDC5 cells were plated at a density of 6 × 10^4^/well in 6-multiwell plastic plates (Corning, New York, NY, USA). Cells were sub-cultured at 70–80% confluence using differentiation medium, which is identical to maintenance media with the addition of 1% ITS. The differentiation medium was changed every other day from day 2 to 14. After differentiation for 14 days, cells were incubated in ITS free medium containing 1% FBS for 24 h and then stimulated with leptin for an additional 24 or 48 h.

### Small interfering RNA-mediated silencing of endogenous expression of ERK1 and ERK2 in ATDC5 cells

Expression of either ERK1 or ERK2 was specifically silenced in ATDC5 cells using small interfering SiRNA purchased from Cell Signaling Technology. SiRNA complexes were prepared and transfection was performed using Lipofectamine 2000 (Invitrogen) according to the manufacturer's instructions. Twenty-four hours after transfection, the cells were lysed and the expression of either ERK1 or ERK2 was assayed by western blot.

### Alcian blue staining

ATDC5 cells were cultured for 4, 7, 14, or 21 days. Cells were rinsed twice with phosphate-buffered saline (PBS), fixed with 4% paraformaldehyde for 15 min, and then stained for 30 min with 1% Alcian blue 8GS (Sigma, St. Louis, MO, USA). The stained cells were washed with PBS three times and then photographed. Experiments were performed in triplicate.

### Western blot analysis

After the treatments, the harvested cells were washed with PBS twice and lysed on ice for 30 min with whole-cell extract RIPA lysis buffer (Thermo Fisher cat:89900). The concentration of protein was quantified using a BCA protein assay (Thermo Fisher cat:23229); equal amounts of protein samples (20 μg) were separated with 12% sodium dodecyl sulfate-polyacrylamide gel electrophoresis and transferred onto nitrocellulose membranes (Beyotime). Immune complexes were formed by incubation with the primary rabbit antibodies against phospho-ERK1/2 (Thr202/Thr204), total-ERK1/2, phospho-p38 MAPK (Thr180/Tyr182), antitotal-p38 MAPK, phospho-AKT (Ser473), AKT, and a primary antibody against β-actin. Blots were washed and incubated for 1 h with a secondary antibody at 1:2000 dilution. Immunoreactive protein bands were detected using an Odyssey scanning system (SYSTEM/Manufacturer Info). The protein expression levels of the kinases were normalized against β-actin.

### Quantitative real-time polymerase chain reaction

Total RNA was isolated using Trizol (Invitrogen, Carlsbad, CA, USA). A spectrophotometer was used to detect the concentration and purity of the RNA. cDNA was obtained by reverse transcriptase (Takara, Japan). Quantitative real-time PCR (RT-PCR) assays were performed on the ABI Prism 7500 Fast System (Applied Biosystems, Carlsbad, CA, USA) using the standard curve method. The following primers were used:

Collagen type II: 5′-GTCCTGAAGGTGCTCAAG GTTCTC-3′; 5′-AGGAATACCATCAGTCCCTGGGTTA-3′; Collagen type X: 5′-AGAACGGCACGCCTACGAT-3′, 5′-CTGTGAGCTCCATGATTGCA-3′;

β-actin (*Actb*): 5′-CATCCGTAAAGACCTCTATGCCAAC-3′, 5′-ATGGAGCCACCGATCCACA-3′;

Leptin (*Lep*): 5′-TGTGAAAAGGACAAAGGAG TTG-3′, 5′-GCTCAGCAATATGCCAACAA-3′;

Osteocalcin *(Bglap)*: 5′-CAGACCTAGCAGACAC CATGA-3′, 5′-CTGCCAGAGTTTGGCTTTAGG-3′

### Statistical analysis

Data are presented as the means ± standard deviation from at least three separate experiments. The independent two-sample t-test was used to draw a comparison between groups. All tests were two-tailed and the significance level was set at *P* < 0.05. The statistical analyses were performed using SPSS 19.0 software (SPSS Inc., Chicago, IL, USA).
